# Efficient Portable Urea Biosensor Based on Urease Immobilized Membrane for Monitoring of Physiological Fluids

**DOI:** 10.3390/biomedicines8120596

**Published:** 2020-12-11

**Authors:** Jee Young Kim, Gun Yong Sung, Min Park

**Affiliations:** 1 Cooperative Course of Nano-Medical Device Engineering, Hallym University, Chuncheon, Gangwon-do 24252, Korea; jyoung@hallym.ac.kr (J.Y.K.); gysung@hallym.ac.kr (G.Y.S.); 2 Integrative Materials Research Institute, Hallym University, Chuncheon, Gangwon-do 24252, Korea; 3 Major in Materials Science and Engineering, Hallym University, Chuncheon, Gangwon-do 24252, Korea

**Keywords:** urea biosensor, real-time monitoring, urease immobilization, disuccinimidyl cross-linker, flow system

## Abstract

Numerous studies have addressed the utilization of glutaraldehyde (GA) as a homobifunctional cross-linker. However, its applicability has been impeded due to several issues, including the tendency of GA molecules to undergo polymerization. Herein, a portable urea biosensor was developed for the real-time monitoring of the flow of physiological fluids; this was achieved by using disuccinimidyl cross-linker-based urease immobilization. Urease was immobilized on a porous polytetrafluoroethylene (PTFE) solid support using different disuccinimidyl cross-linkers, namely disuccinimidyl glutarate (DSG), disuccinimidyl suberate (DSS) and bis-N-succinimidyl-(pentaethylene glycol) ester (BS(PEG)_5_). A urease activity test revealed that DSS exhibited the highest urease immobilizing efficiency, whereas FT-IR analysis confirmed that urease was immobilized on the PTFE membrane via DSS cross-linking. The membrane was inserted in a polydimethylsiloxane (PDMS) fluidic chamber that generated an electrochemical signal in the presence of a flowing fluid containing urea. Urea samples were allowed to flow into the urea biosensor (1.0 mL/min) and the signal was measured using chronoamperometry. The sensitivity of the DSS urea biosensor was the highest of all the trialed biosensors and was found to be superior to the more commonly used GA cross-linker. To simulate real-time monitoring in a human patient, flowing urea-spiked human serum was measured and the effective urease immobilization of the DSS urea biosensor was confirmed. The repeatability and interference of the urea biosensor were suitable for monitoring urea concentrations typically found in human patients.

## 1. Introduction

Biosensors are analytical devices that detect biomolecules, comprising three parts, namely a molecular recognition layer, a transducer and a signal generator [1]. Molecular recognition layers have been developed for the specific capture of various biomolecules, including antibodies, aptamers, enzymes and receptors [2,3,4,5,6]. Enzyme-based biosensors are powerful analytical devices that use an enzyme to capture analytes and generate a measurable recognition signal [7,8]. Urease is an enzyme that hydrolyzes urea and is widely used for the detection of urea. Mammals, including humans, produce urea as the main end-product of protein metabolism, and its levels are directly related to the protein intake and nitrogen metabolism [9]. As urea is excreted by the kidneys, it is an important biomarker of kidney dysfunction [10,11].

Various biosensors based on urease have been developed using electrochemistry, optics, and piezoelectricity [12,13,14]. Electrochemical urea biosensors are most widely applied due to their high sensitivity and simplicity [15]. Although the functions of various electrochemical urea biosensors are based on amperometry, voltammetry, and impedance spectroscopy, the practicality of these devices remains an issue [16,17,18,19]. The most common disadvantages are a narrow dynamic range and the need for static measuring conditions [12], because biosensing under flow conditions is essential in the real-time monitoring of a living physiological sample [20,21].

The sensitive molecular recognition layer of an enzyme-based biosensor is produced by immobilizing an enzyme on a transducer or solid support, and this step is vital to the functioning of the device [22,23]. Various immobilization methods have been used in previous studies, including physical adsorption, enzyme entrapment and cross-linking [24]. Covalent immobilization by cross-linking is considered to produce the strongest bonding between the solid support and the enzyme and is associated with minimal leakage [25]. Glutaraldehyde (GA; all abbreviations used in this study are listed in Table 1) is a homobifunctional cross-linker that has been widely applied in the immobilization of enzymes, the fixation of biological samples and the development of biocatalysts due to its simple and powerful reactivity with amino groups of proteins [26]. However, several issues are associated with the use of GA, including the easy polymerization of GA molecules, the instability of aldehyde groups and the reversibility of amine–aldehyde reactions [26,27]. Bifunctional amine active N-hydroxysuccinimide esters with crosslinkers have been used for improved stability [25]. The reactive functional groups and the spacing length between the functional ends are important in enzyme immobilization [28]. Changes in the length of a functional group in a cross-linker will affect the activity of the immobilized enzyme and thus the sensitivity of the biosensor [29].

In this study, we aimed to develop a portable biosensor based on a urease-immobilized membrane. For the development of an efficient biosensor, effective urease immobilization was carried out using disuccinimidyl cross-linkers. The activity of immobilized urease on the porous membrane was tested and the performance of the urea biosensor was also tested. We further investigated the efficiency of this device to monitor urea-spiked human serum under flow conditions and compared it to that using the more commonly used GA.

## 2. Experimental Section

### 2.1. Materials

Urease derived from jack bean (*Canavalia ensiformis*), urea, human serum, GA, ammonium carbamate, disodium hydrogen phosphate, potassium dihydrogen phosphate, uric acid, glucose, glycine, sodium chloride and sucrose was obtained from Merck (Darmstadt, Germany). Disuccinimidyl glutarate (DSG), disuccinimidyl suberate (DSS) and bis-N-succinimidyl-(pentaethylene glycol) ester (BS(PEG)_5_) were obtained from Thermo Fischer Scientific (Waltham, MA, USA). A urea assay kit was obtained from BioAssay Systems (Hayward, CA, USA). Phosphate buffered saline (PBS) was obtained from LPS solution (Daejeon, Korea). A porous polytetrafluoroethylene (PTFE) membrane with a pore size of 1.00 µm was obtained from Advantec MFS (Dublin, CA, USA).

### 2.2. Parylene Coating of the PTFE Membrane and Urease Immobilization

The surface of the PTFE membrane and a 96-well microplate were modified with active amines. An amine-functionalized poly (p-xylylene) (parylene-A) thin film coating with a thickness of 100 nm was deposited using the following procedure. Parylene-A was evaporated at 160 °C and pyrolyzed at 650 °C to yield the highly reactive amine-functionalized p-xylene radical. The parylene-A film was uniformly deposited on the PTFE membrane and microplate at room temperature. The amine-functionalized surface was converted to amine active groups, namely aldehyde and succinimidyl, by applying a 1-mM solution of crosslinking agents (GA, DSS, DSG and BS(PEG)_5_) for 1 h. Urease (48 mg/mL) was immobilized by covalently bonding the free amine groups in urease to the amine active groups on the surface of the PTFE membrane and microplate.

### 2.3. Urease Urease Activity Assay

A commercial urea assay kit was used to measure the urea concentrations in the samples. Activity testing of the microplate involved adding 16.67 mM urea to the microplate well and incubating for 1 h at 25 °C. Activity testing of the PTFE membrane involved dipping the membrane in 16.67 mM urea for 1 h at 25 °C while shaking. The urease-immobilized microplate and PTFE membrane hydrolyzed the urea solutions. A 5-μL sample of the hydrolyzed urea solution was transferred to an unmodified 96-well microplate and treated with 200 µL phthalaldehyde reagent from the urea assay kit for 20 min. The activity of the urease was measured using colorimetry based on optical density (OD) at 520 nm.

### 2.4. Urease Configuration of the Flow System and the Urea Biosensor

**Figure 1:**
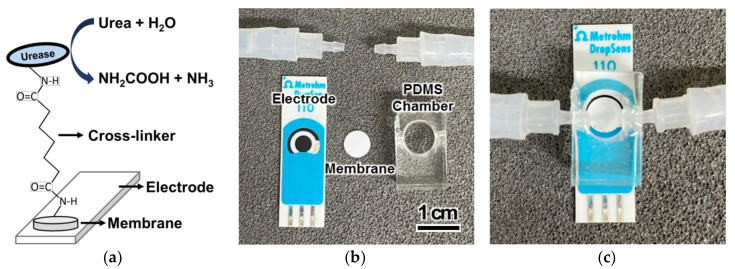
Schematic diagram of (**a**) the configuration of the urease-immobilized membrane; the configuration of the fluidic compartment of the urea biosensor (**b**) before and (**c**) after assembly.

A cylindrical fluidic chamber with a diameter of 8 mm and a height of 5 mm was produced using polydimethylsiloxane (PDMS) (Figure 1). Eight urease-immobilized membranes were placed in the chamber, while an aminated screen-printed glassy carbon electrode, with a diameter of 4 mm, (Metrohm, Herisau, Switzerland) was positioned beneath the chamber (Figure 1b). The chamber was covered with 2 mm of PDMS and the inlet and outlet tubes were inserted into the sides of the chamber. The chamber was placed in a housing that was 2 cm wide, 3.5 cm long and 1.5 cm high. The assembled chamber was inserted into the housing to avoid leakage [21].

### 2.5. Urease Electrochemical Measurements in the Flow Condition

The urea sample flowed into the fluidic chamber via the inlet tube at a constant flow rate of 1.0 mL/min, controlled using a peristaltic pump (ISMATEC, Wertheim, Germany), and out of the chamber to a waste bottle via the outlet. The electrochemical measurement of urea under flow conditions was conducted based on chronoamperometry at a constant voltage of 1.0 V, in which a three-electrode was connected to potentiostats (Metrohm, Herisau, Switzerland) to measure the electrical current produced during the hydrolysis of urea by urease.

## 3. Results and Discussion

### 3.1. Immobilization of Urease on the Solid Support

**Figure 2:**
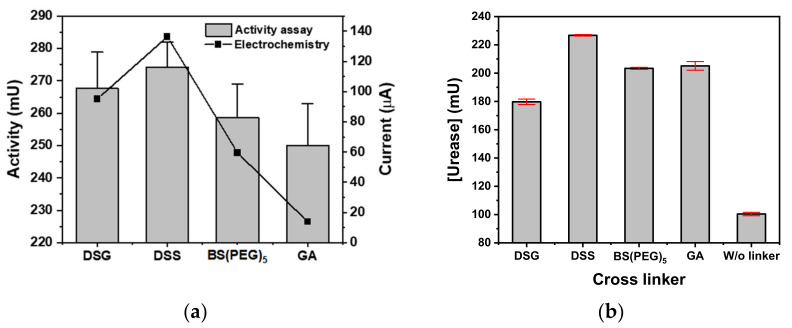
Urease activity of the cross-linker urease-immobilized parylene-A coated (**a**) microplate and (**b**) PTFE membrane measured using a commercial urea assay kit (gray bar) and electrochemistry (■).

Urease was covalently immobilized on a parylene-A-coated PTFE membrane with disuccinimidyl cross-linkers of various spacing, namely DSG, DSS and BS(PEG)_5_, and compared to the immobilization of urease using GA. The atomic spacing of DSG, DSS and BS(PEG)_5_ was 7.7, 11.4 and 21.7 Å, respectively, where the chain length and cross-linker flexibility of DSG was the lowest and BS(PEG)_5_ was the highest. The spacing structure of DSG and DSS was an alkyl chain, whereas BS(PEG)_5_ contained a chain of five flexible ether bonds. All disuccinimidyl cross-linkers exhibited higher urease activity than GA when applied to the commercial urea assay microplate—the urease activities of DSG, DSS, BS(PEG)_5_ and GA were 267.6, 274.2, 258.7 and 250.1 mU, respectively (Figure 2a). The urease-immobilized microplate using DSS exhibited the highest activity. The electrochemistry current values of the DSG, DSS, BS(PEG)_5_ and GA urease-immobilized microplates were 95.1, 136.4, 59.5 and 13.8 µA, respectively. Similarly, all of the disuccinimidyl cross-linkers exhibited higher urease activity than GA, with DSS producing the highest current. DSS exhibited the highest urease immobilizing efficiency across both measures of urease activity, namely the commercial urea assay and the electrochemical test.

The parylene-A coated PTFE membranes (8 mm diameter) were used in urea biosensors. The immobilized urease was compared to a control of GA and a negative control of urease immobilized without the use of a cross-linker (physical adsorption). The activities of the urease-immobilized porous membranes produced using DSG, DSS, BS(PEG)_5_, GA and no cross-linker were 179.7, 226.8, 203.4, 205.1 and 100.4 mU, respectively (Figure 2b). The urease activity of the covalently bonded urease (DSG, DSS, BS(PEG)_5_ and GA) was at least 1.8 times higher than that of the physically adsorbed urease (no cross-linker). DSS continued to exhibit the highest activity and was 26.8%, 11.5% and 10.6% higher than DSG, BS(PEG)_5_ and GA, respectively.

Among the disuccinimidyl cross-linkers, BS(PEG)_5_ had the longest chain length and flexibility, which provides the immobilized enzyme with more freedom and allows for higher enzymatic activity. Thus, BS(PEG)_5_ was expected to have the highest immobilizing efficiency. However, the urease activity was related to the cross-linker immobilization efficiency, wherein the long and flexible cross-linkers of BS(PEG)_5_ had a higher probability of reacting with their own active amine groups. This neutralized the activity of the cross-linkers, thus decreasing the number of enzyme immobilization sites. By comparing the urease immobilization of DSG, DSS and BS(PEG)_5_ on an amine-activated solid surface, DSS had the highest immobilizing efficiency.

### 3.2. Analysis of the Urease-Immobilized Membrane Produced by DSS Cross-Linking

**Figure 3:**
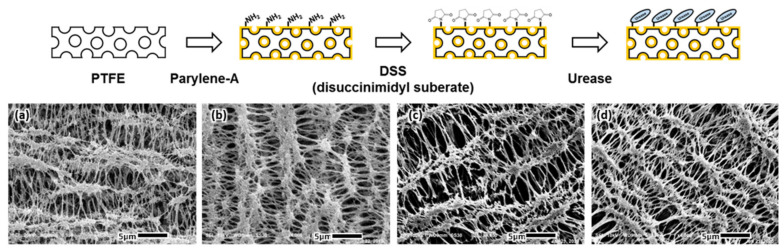
The microstructure of the PTFE membrane during the urease immobilization process, namely the (**a**) bare, (**b**) parylene-A coated, (**c**) DSS treated, and (**d**) urease-immobilized PTFE membrane.

A commercial PTFE membrane with a porous structure (average pore size of 1 µm) was selected as the solid support. The porous structure provided a larger surface area and, in turn, more urease immobilizing sites to produce an electrochemical signal under flow conditions. The microstructure of the membrane was observed during urease immobilization using scanning electron microscopy (SEM) (Figure 3). The porous microstructure of the PTFE membrane was not affected by the parylene-A coating that was used to surface-modify the membrane with active amine groups. The porous microstructure was maintained during further treatment with the DSS cross-linker and urease enzyme. Urease immobilization did not affect the porous microstructure, allowing the urease-immobilized membranes to effectively generate an electrochemical signal under flow conditions.

The atomic composition of the PTFE membrane was analyzed using energy dispersive spectrometry (EDS) in SEM. The membrane included alkyl chains saturated with fluorine and was composed of only carbon (40.7%) and fluorine (59.3%). The addition of the parylene-A layer added nitrogen (6.4%) and adjusted the amounts of carbon (32.3%) and fluorine (61.3%). DSS treatment introduced oxygen (10.7%), attributed to the succimidyl groups of DSS, and adjusted the amounts of nitrogen (6.9%), carbon (41.4%) and fluorine (40.9%). Immobilization of urease increased the atomic ratios of nitrogen and oxygen and resulted in a final atomic distribution of 11.9% oxygen, 7.3% nitrogen, 30.7% carbon and 50.1% fluorine. Urease contains relatively high amounts of nitrogen and oxygen compared to other organic polymers, and this change in atomic composition indicated that urease was successfully immobilized.

**Figure 4:**
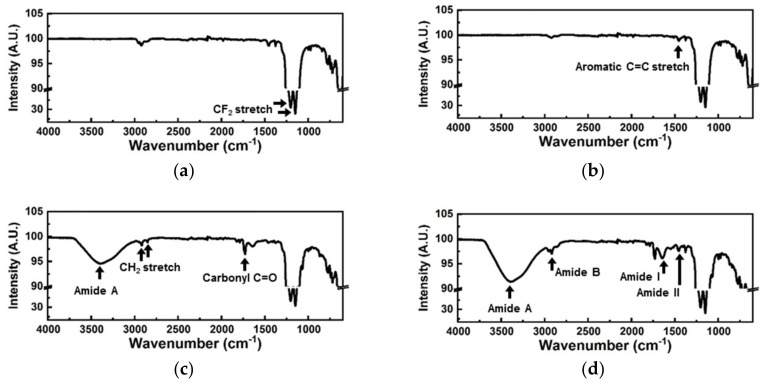
Fourier-transform infrared (FT-IR) spectra during the urease immobilization process, where the (**a**) the PTFE membrane was sequentially treated with (**b**) parylene-A, (**c**) DSS and (**d**) urease.

The FT-IR spectrum of the bare PTFE membrane exhibited strong CF2 stretching peaks at 1201 nm and 1153 nm (Figure 4a), whereas the spectrum of the parylene-A coated PTFE membrane included an aromatic C=C stretching peak (Figure 4b). Parylene-A has a phenyl group in its backbone, and the aromatic peak observed indicated that the PTFE membrane was successfully coated with parylene-A. DSS treatment gave rise to amide A (3400 nm) and carbonyl C=O (1730 nm) peaks, attributed to the succinimidyl groups (Figure 4c). CH2 stretching peaks from the alkyl group in the spacing structure of DSS were also observed at 2920 and 2850 nm. These four peaks indicated that DSS was successfully immobilized on the amine-activated parylene-A-coated PTFE membrane. Various amide peaks, attributed to the peptide bonds in urease, were observed after urease immobilization, including amide A (3400 nm), amide B (2920 nm), amide I (1640 nm) and amide II (1455 nm), indicating that urease was immobilized on the PTFE membrane (Figure 4d).

### 3.3. Urea Biosensing Using Urease-Immobilized Membranes

**Figure 5:**
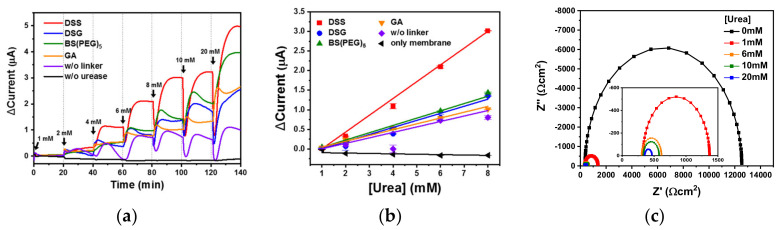
(**a**) Real-time monitoring of urea-spiked phosphate buffered saline (PBS) using the urea biosensors under flow conditions and (**b**) the biosensor response and (**c**) Nyquist plots according to changing urea concentration.

Eight urease-immobilized membranes were inserted into a PDMS fluidic chamber to measure the signal of urea-spiked PBS using chronoamperometry at a constant voltage (Figure 1) [21]. Urea was hydrolyzed to carbamic acid and ammonia by urease as follows: (NH_2_)_2_CO + H_2_O → NH_2_COOH + NH_3_. The unstable carbamic acid subsequently formed hydrazine (N_2_H_4_) via dimerization and decarboxylation, and the electrode was oxidized by the free nitrogen from hydrazine [30]. Real-time monitoring using the urea biosensor was trialed by flowing various concentrations of urea-spiked PBS into the urea biosensor at 1.0 mL/min. The real-time electrochemical signal from the various cross-linker treated membranes was measured using chronoamperometry (Figure 5a). The DSG, DSS and BS(PEG)_5_ urea biosensors showed increased sensor responses with increasing urea concentration, and all produced a higher current than the GA (orange)-treated membrane. The DSS urea biosensor (red) exhibited the highest current values across all urea concentrations, as expected from the microplate (Figure 2a) and membrane (Figure 2b) activity tests. The urea biosensor response was proportional to the immobilized urease activity—higher urease activity can increase the sensitivity of the biosensor. Especially in the DSS urea biosensor, the signal from the lowest urea concentration (1 mM) was detectable. In the case of physically adsorbed urease, the urea biosensor (violet) produced an unstable signal that did not increase at urea concentrations above 6 mM. Signal saturation at a relatively low urea concentration indicated that the immobilization capacity of the physical adsorption was much lower than covalent immobilization. Thus, covalent immobilization of urease was more suitable for the real-time monitoring of urea under flow conditions. The negative control, a parylene-A coated PTFE membrane without urease treatment (black), exhibited no electrochemical signal, indicating that an electrochemical signal was only generated in the presence of urease, and urea alone did not generate noise.

The current was plotted according to urea concentration (1 to 8 mM) and all of the urea biosensors, namely DSG(●), DSS(■), BS(PEG)_5_(▲), GA(▼) and physical adsorption (◆), exhibited good linearity with R-squared values of 0.97, 0.99, 0.99, 0.99 and 0.99, respectively (Figure 5b). The negative control exhibited poor linearity with an R-squared value of 0.66. The limit of detection (LOD) was calculated from the linear plot using three-sigma confidence intervals and was found to be 1.3, 1.1, 1.3, 1.5 and 1.6 mM for the DSG, DSS, BS(PEG)_5_, GA and physical adsorption urea biosensors, respectively. Urea in human blood typically ranges between 1.2 and 3.3 mM [31,32], and levels below this range in serum are uncommon [33]. Thus, the LOD of the DSS urea biosensor was suitable for monitoring the urea concentration in clinical samples. With the exception of physical adsorption, the signal of all of the covalently immobilized membranes increased until a urea concentration of 20 mM was reached, indicating that the saturation concentrations of these urea biosensors were above 20 mM. During renal failure, the blood urea concentration is typically above 10 mM [34]. Thus, when the urea concentration in physiological fluid is 10 mM or higher, the urea biosensor should sound an alarm to prompt further medical intervention. The dynamic ranges of the urea biosensors based on the covalently immobilized membranes were from their respective LOD to 20 mM. Therefore, the dynamic ranges of these biosensors were appropriate for the monitoring of urea in physiological fluids under flow conditions [35]. LODs from biosensors that monitor the urea concentration in flow conditions are compared in Table 2, and the DSS-based urea biosensor showed improved LOD in comparison with previous studies [21,22]. The sensitivities of the DSG, DSS, BS(PEG)_5_, GA and physical adsorption urea biosensors were 1.5, 3.4, 1.6, 1.1 and 0.9 mA M^−1^ cm^−2^, respectively. The DSS cross-linker produced a biosensor that was three times more sensitive than that using GA, likely due to the superior immobilizing efficiency of DSS. Using the DSS urea biosensor, Nyquist plots according to urea concentration (0 to 20 mM) were obtained in a frequency range from 10^−2^ to 10^5^ Hz in the redox probe, [Fe(CN)_6_]^4−/3−^. As shown in Figure 5c, the electron-transfer resistance (R_ct_) values of 0,1, 5, 10 and 20 mM urea were measured to be 12.1, 1.0, 0.3, 0.2 and 0.1 kΩ, respectively. This means that the R_ct_ values were decreased by the increase of urea concentration due to the hydrolysis of urea by urease.

**Figure 6:**
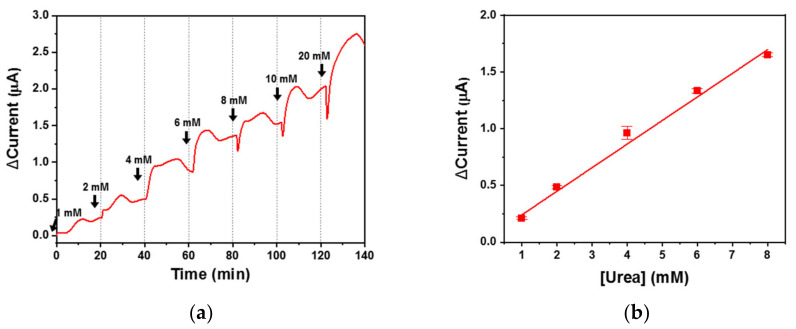
(**a**) Real-time monitoring of urea-spiked human serum using the urea biosensors under flow conditions and (**b**) the biosensor response according to changing urea concentration with linear fitting.

DSS was selected as the most effective cross-linker for urease immobilization, and the DSS urea biosensor was used to monitor urea-spiked human serum. The urea sample flowed into the biosensor at 1.0 mL/min and a real-time electrochemical signal was measured using chronoamperometry (Figure 6a). Despite the thousands of proteins present in serum, a complex physiological fluid, the biosensor responded favorably and reported an increased signal with increasing urea concentration. Although the signal was less stable for the urea samples than the PBS samples, there were clear signal differences across the urea concentration range. Thus, the DSS urea biosensor can be used for urea monitoring in physiological fluids under flow conditions.

The current was plotted according to urea concentration (1 to 8 mM) and the DSS urea biosensor exhibited good linearity, with an R-squared value of 0.99 when applied to serum (Figure 6b). The LOD was 1.2 mM, thus giving the DSS urea biosensor a dynamic range of 1.2 to 20 mM, which is suitable for real-time monitoring of patient sera under flow conditions. The sensitivity was 1.6 mA M^−1^ cm^−2^ and was higher than that of a GA urea biosensor when applied to both PBS and serum. The DSS urea biosensor offered more sensitivity with respect to urea monitoring in serum than widely used enzyme-immobilized biosensors. All measures of performance for the DSS urea biosensor are listed in Table 3.

### 3.4. Repeatability and Interference Analysis

**Figure 7:**
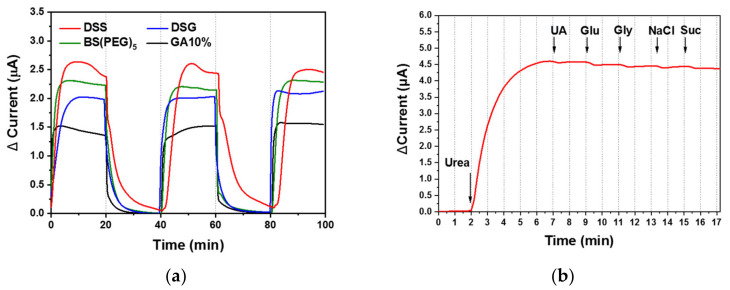
(**a**) The DSS urea biosensor was subjected to (**a**) repeatability testing (in triplicate) of urea biosensor performance, in which 10 mM urea-spiked PBS solution flowed into the biosensor for 20 min, followed by pure PBS for 20 min; and (**b**) interference testing with various interfering species (2 µM uric acid (UA), 30 µM glucose (Glu), 2 µM glycine (Gly), 3 mM sodium chloride (NaCl) and 50 µM sucrose (Suc)), in which 10 mM urea PBS solution flowed into the biosensor, followed by 10 mM urea PBS solution with the interfering substance.

The repeatability of the urea biosensor performance was validated by continuously monitoring urea in triplicate, in which 10 mM urea-spiked PBS flowed into the biosensor for 20 min, followed by pure PBS for 20 min (Figure 7a). The electrochemical signal increased in response to the 10-mM urea solution and decreased with the pure PBS (blank sample) across all tests. The relative standard deviations (RSDs) of the triplicate measurements were 3.7%, 0.7%, 3.2% and 6.0% for the DSG (blue), DSS (red), BS(PEG)_5_ (green) and GA (black) urea biosensors, respectively. The RSDs of the disuccinimidyl-based cross-linker (DSG, DSS and BS(PEG)_5_) biosensors were below 5%, whereas the widely used GA cross-linker exhibited the highest RSD value. The DSS urea biosensor exhibited a particularly low RSD value (<1%), which confirmed that urease immobilization using DSS allowed for sensitive and reliable real-time monitoring of urea under flow conditions.

Interference was tested by introducing various organic and ionic species in the 10 mM urea-spiked PBS solution, including 2 µM uric acid, 30 µM glucose, 2 µM glycine, 3 mM sodium chloride and 50 µM sucrose. The 10 mM urea solution flowed through the DSS urea biosensor, followed by the 10 mM urea solution containing the interfering substance. There was minimal change in chronoamperometric response. The addition of uric acid, glucose, glycine, sodium chloride and sucrose caused a 0.3%, 2.3%, 3.4%, 3.0%, and 4.4% fluctuation in signal, respectively (Figure 7b). Interference values below 5% demonstrated the good selectivity of the DSS urea biosensor for urea, emphasizing the potential for its use for monitoring urea concentration in flowing physiological fluid with high sensitivity, repeatability and selectivity.

## 4. Conclusions

A portable urea biosensor (2 cm wide, 3.5 cm long and 1.5 cm high) was fabricated based on a urease-immobilized membrane that generated an electrochemical signal in the presence of a flowing fluid containing urea. Urease was immobilized effectively using three types of disuccinimidyl-based cross-linkers, namely DSG, DSS and BS(PEG)_5_, which were compared with the more widely used GA. Urease activity assays were conducted on a microplate and membrane, and DSS exhibited the highest urease immobilization efficiency. The cross-linker covalently immobilized urease enzymes on the parylene-A coated porous membrane, as demonstrated using FT-IR analysis. The urease-immobilized membranes were placed on an electrode and inserted into a PDMS fluidic chamber. A 10-mM urea-spiked PBS solution flowed through the urea biosensor and the signal was measured using chronoamperometry. The sensitivities of the DSG, DSS, BS(PEG)_5_ and GA urea biosensors were 1.5, 3.4, 1.6 and 1.1 mA M^−1^ cm^−2^, respectively. Thus, DSS was selected for further testing of urea-spiked human serum as a demonstration of the biosensors’ applicability to living patients. The DSS urea biosensor was sufficiently sensitive in human serum (1.6 mA M^−1^ cm^−2^) and showed the potential for real-time monitoring of urea under flow conditions. The repeatability and selectivity of the monitoring was high, with an RSD below 1% and interference from different organic and ionic species below 5%. The DSS cross-linking-based urea biosensor was suitable for the monitoring of urea concentrations in flowing physiological fluids with superior sensitivity, repeatability and selectivity in comparison with GA cross-linking-based urea biosensors. This urea biosensor is suitable for portable applications including point-of-care testing, real-time monitoring systems (e.g., portable dialysis systems) and in artificial kidneys.

## Figures and Tables

**Table 1:** Abbreviations used in this study.

Abbreviation	Expansion
GA	glutaraldehyde
PTFE	polytetrafluoroethylene
DSG	disuccinimidyl glutarate
DSS	disuccinimidyl suberate
BS(PEG)_5_	bis-*N*-succinimidyl-(pentaethylene glycol) ester
PDMS	polydimethylsiloxane
Parylene-A	amine-functionalized poly (*p*-xylylene)
OD	optical density
LOD	limit of detection
RDS	relative standard deviation

**Table 2:** Comparison of limits of detection (LODs) of biosensors that monitor the urea concentration in flow conditions. Listed LOD was measured at a flow rate of 1.0 mL/min.

Urease Immobilizing Substrate	Cross-Linker	LOD	Reference
Silk fibroin	GA	-	[20]
Porous PTFE	GA	4 mM	[21]
Porous PTFE	DSS	1.1 mM	This work

**Table 3:** Measures of the performance of the DSS-based urea biosensor.

Factor	Performance
LOD	1.1 mM (PBS) 1.2 mM (human serum)
Sensitivity	3.4 mA M^−1^ cm^−2^ (PBS) 1.6 mA M^−1^ cm^−2^ (human serum)
Dynamic range	1.1–20 mM (PBS) 1.2–20 mM (human serum)
